# Drought and Salinity Stress Responses and Microbe-Induced Tolerance in Plants

**DOI:** 10.3389/fpls.2020.591911

**Published:** 2020-11-13

**Authors:** Ying Ma, Maria Celeste Dias, Helena Freitas

**Affiliations:** University of Coimbra, Centre for Functional Ecology, Department of Life Sciences, Coimbra, Portugal

**Keywords:** drought, salinity, photosynthesis, osmotic adjustment, metabolic regulation, plant-microbe interactions, phytohormonal regulation, plant adaptations

## Abstract

Drought and salinity are among the most important environmental factors that hampered agricultural productivity worldwide. Both stresses can induce several morphological, physiological, biochemical, and metabolic alterations through various mechanisms, eventually influencing plant growth, development, and productivity. The responses of plants to these stress conditions are highly complex and depend on other factors, such as the species and genotype, plant age and size, the rate of progression as well as the intensity and duration of the stresses. These factors have a strong effect on plant response and define whether mitigation processes related to acclimation will occur or not. In this review, we summarize how drought and salinity extensively affect plant growth in agriculture ecosystems. In particular, we focus on the morphological, physiological, biochemical, and metabolic responses of plants to these stresses. Moreover, we discuss mechanisms underlying plant-microbe interactions that confer abiotic stress tolerance.

## Introduction

Plant growth, development, productivity, and resistance to climatic stresses are currently the major topics of interest for agriculture and plant-based biotechnologies. Both biotic (e.g., phytopathogens) and abiotic stresses (e.g., drought, salinity, flood, storm, and extreme temperatures) cause enormous losses in agricultural production (Fraire-Velázquez and Balderas-Hernández, 2013). In the past decades, tremendous progress has been made in understanding the mechanisms underlying plant resistance/tolerance to individual biotic and/or abiotic stresses. Moreover, plant responses to various stresses and their positive or negative impacts on plant growth have been comprehensively studied (Abuqamar et al., 2009). However, plants existing in nature must simultaneously cope with diverse and interacting stresses that generally occur simultaneously or in sequence (Pandey et al., 2015). It is well known that drought and salinity are the two foremost abiotic stresses that reduce the global productivity of major crops (Kaushal and Wani, 2016; Singh et al., 2018). So far, our current knowledge about the key processes involved in plant adaptations to abiotic stress conditions is still very limited. Therefore, there is a need to understand the mechanisms of plant tolerance/adaptation and mitigation strategies to abiotic stresses.

**Figure 1:**
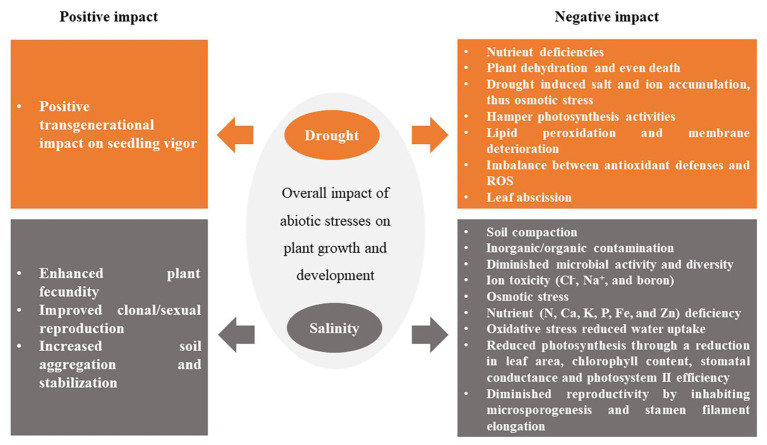
Impacts of drought and salinity on plant growth. The arrows point to an effect and the colors are the origins (cause). For example, from Drought → its effects on plants, which the arrows and boxes are orange; Salinity → its effects on plants, which the arrow and boxes are colored dark gray.

In the present review, we focus on recent advances in understanding the mechanisms involved in plant response/adaption to the selected environmental stresses (e.g., drought and salinity) at the morphological (e.g., root morphology, plant growth, and yield), physiological and biochemical (e.g., photosynthesis, pigment, osmotic adjustment, and antioxidants), and metabolomic (e.g., metabolite adjustments) levels, as well as plant-microbe interactions that confer abiotic stress tolerance in plants (**Figure 1**).

## Plant Adaptation to Drought and Salinity Stress

**Figure 2:**
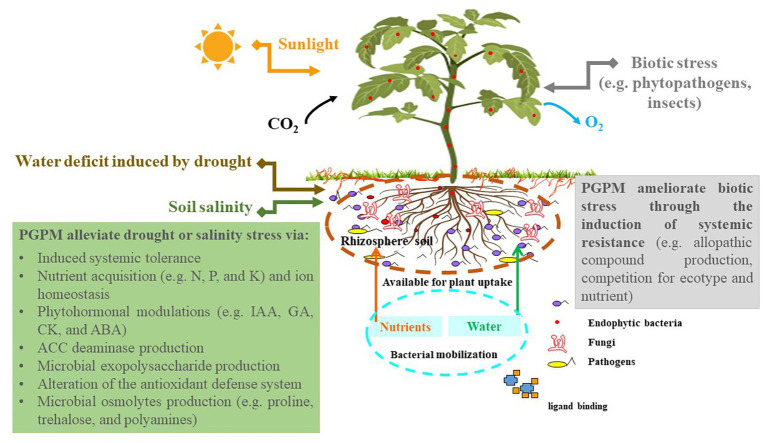
Plant-plant growth-promoting microorganism (PGPM) interactions confer abiotic stress tolerance. PGPM, plant growth-promoting microorganisms; IAA, indole-3-acetic acid; GA, gibberellins; CK, cytokinins; ABA, abscisic acid; ACC, 1-amino-cyclopropane-1-carboxylic acid; N, nitrogen; P, phosphate; K, potassium.

Plant physiology is significantly affected by abiotic/climatic stresses. It is well known that climate change and environmental extremes induce and enhance the impact of abiotic stresses (particularly drought and salinity) on plant fitness and performance (Kaushal and Wani, 2016). The positive and negative impacts of drought and salinity on plant growth and development are summarized in Figure 2. Besides all the negative effects induced by drought and salinity on plant performance, to some extent, drought or salinity may result in some positive effects on plants (Raza et al., 2019). For instance, Hatzig et al. (2018) found that drought stress showed a positive transgenerational impact on seedling vigor of *Brassica napus*. They attributed this to heterotic effects, the altered reservoir of seed storage metabolites, and inter-generational stress memory formed by stress-induced changes in the epigenome of the seedling. Salinity at certain concentrations may also cause an increase in clonal/sexual reproduction, thus improving plant fecundity (Van Zandt et al., 2003). This effect strongly depends on the genotypes/cultivar and plant developmental stage.

To meet the needs of developing sustainable agriculture and improving food safety, it is necessary to grow stress-tolerant plants and understand the mechanisms underlying their tolerance. In general, plant responses to abiotic stresses vary in morphology, physiology, biochemistry, and metabolism, which will be discussed in the following sections.

## Plant Morphological Responses to Drought and Salinity Stress

Since water is vital to life on earth, drought or osmotic stress may significantly affect many aspects of plant morphology and physiology (Suwa et al., 2006; Rahdari and Hoseini, 2012). Exposure of plants to drought stress generally results in a significant reduction of growth and yield of crops such as *Hordeum vulgare*, *Oryza sativa*, *Triticum aestivum*, *Zea mays* (Kamara et al., 2003; Samarah, 2005; Rampino et al., 2006; Lafitte et al., 2007) due to low humidity in plants, high intensity of sunlight, high temperature caused by drought with enhanced respiration, photosynthesis, and enzyme activity in plants (Fathi and Tari, 2016). In the early phase of drought stress, gradual water depletion causes a decrease in shoot growth, whereas root growth is maintained, resulting in an enhanced root/shoot ratio (Bogeat-Triboulot et al., 2007). Plants subjected to moderate drought normally show a slight change in their growth pattern, along with only a small increase in the root mass fraction (RMF) calculated as the proportion of plant dry mass in roots. The plants seem to maintain their aboveground growth and, therefore, their competitiveness for aboveground resources, as long as possible under moderate drought. In contrast, plants exposed to severe drought, Poorter et al. (2012) reported that when the biomass is reduced by more than 50% compared with control plants, respond with a strong increase in RMF, which can be largely attributed to a decrease in the growth of the stems. Under the moderate or severe drought conditions, the accumulation of salts and ions in soil upper layers leads to osmotic stress and ion toxicity in plants. With an increase in the degree of drought stress, turgor pressure of the plant cells decreases, causing plant cell wall wrinkled and loose. These biophysical responses may eventually reduce the size and number of the leaves as well as fresh weight and water content of plants (Jaleel et al., 2009; Fathi and Tari, 2016). Under mild or moderate drought stress, the roots may change their architecture and allocation of resources (water and nutrients) to avoid dehydration (Smith and De Smet, 2012; Hasibeder et al., 2015). However, under severe drought stress conditions, the roots shrink, and the photosystem II becomes dysfunctional in the leaves (Fathi and Tari, 2016). During soil (low soil moisture) drought and atmospheric aridity (high vapor pressure deficit), the profiles of root exudation (e.g., composition and concentration) may also shift, therefore, influencing the rhizosphere soils properties (Henry et al., 2007; Song et al., 2012). Besides, the availability and plant uptake of nutrients in soils can be affected by drought, since nutrients are carried to the roots by water. Drought stress may further reduce nutrient diffusion and mass flow of water-soluble nutrients (Selvakumar et al., 2012).

Salinity adversely affects seed germination, plant growth, and development, causing significant crop yield losses worldwide (AbdElgawad et al., 2016). As a consequence of salinity stress, seed germination decreases by regulating the abscisic acid (ABA) concentration *via* changes in 9-cis-epoxycarotenoid dioxygenase3/salt tolerant1 (NCED/STO1) expression (Wang et al., 2015). Moreover, salinity stress can regulate the activity of two ethylene biosynthesis enzymes, namely 1-amino-cyclopropane-1-carboxylic acid (ACC) synthase and ACC oxidase. It is well known that ethylene has an essential role in stimulating the dormancy-breakage and seed germination of several plant species by inhibiting ABA functions (Ribeiro and Barros, 2006; El-Maarouf-Bouteau et al., 2015) and can contribute to seed tolerance to salinity (Silva et al., 2014). Therefore, the balance between ethylene and ABA is crucial to modulate seed germination to cope with salinity stress (González-Guzmán et al., 2002). Sibole et al. (2003) reported that stem and petiole growth of *Medicago arborea* and *Melaleuca citrina* was inhibited under salinity stress. Besides, salinity stress may significantly influence carbon and nitrogen metabolism in plants (Obata and Fernie, 2012) and induce alterations in the concentrations of tricarboxylic acid cycle intermediates, sugars, and amino acids in plants to maintain metabolic homeostasis under increasing salt concentrations (Richter et al., 2015; Li et al., 2017).

In recent decades, remarkable advances have been made in various domains of stress physiology, among which long‐ and short-distance signaling plays an essential role in the feed-forward and the feed-back control of photosynthesis in response to drought and salinity. Several studies indicate that signaling may act even before that the cell biophysical alterations occur (Mittler, 2006; Huang et al., 2012). It is well known that signaling pathways causing plants’ responses to various stresses are interconnected at many levels. To adapt to drought and salinity stresses, plants have developed diverse stress-responsive signaling pathways and activated defense mechanisms (Huang et al., 2012). Plants can employ multiple stress perception and signal transduction pathways, which can crosstalk at different steps in the pathways. When drought and salinity occur simultaneously, plants can also exhibit strategic defense responses which could be distinctive from the response to either individual stress (Koussevitzky et al., 2008). Thus, the molecular and metabolic responses to a combination of stresses are unique and cannot be extrapolated from plant response to the individual stress (Mittler, 2006).

## Plant Physiological and Biochemical Response to Drought and Salinity

Early responses to drought and salinity are very similar since both induce water stress that leads to a slowdown in growth, a decrease in stomatal aperture, and a nutrient deficiency (such as K^+^ and Ca^2+^). However, during long term exposure to salt, besides dehydration, plants experience ionic stress, which leads to leaf senescence and photosynthesis impairment (that in turn exerts an additional negative effect on growth; Chaves et al., 2009). When plants are exposed to long-term drought stress, continued root elongation occurs, which may be explained by the plant’s need to reach groundwater (Brunner et al., 2015). While under long-term salinity stress, heavier roots may accumulate higher amounts of chloride. The excessive accumulation of ions, predominantly Na^2+^, affects negatively the photosynthetic components, therefore, reducing enzyme activities and pigment synthesis. These stressful conditions decrease the CO_2_ assimilation rate, and the excess of light absorbed that is not used by the plant can lead to an increase in reactive oxygen species (ROS) production and consequently oxidative stress. Many species possessing salt exclusion mechanisms can prevent salt entry in plant cells, or they can minimize salt concentration in the cytoplasm by compartmentalizing salt in the vacuoles (Khan et al., 2019). These strategies reduce the negative effects on photosynthesis and other metabolic processes. In salinity acclimated plants, some metabolites related to primary metabolism (e.g., carbohydrates, amino acid, and nitrogen) play an important protective role acting as osmolytes (protecting membranes and protein) and ROS scavengers (Sharma et al., 2019).

### Photosynthesis

Photosynthesis is one of the main processes affected by drought and salinity (Chaves et al., 2009). Stomata have two important key functions in plants: one is to regulate transpiration, which supplies plants with nutrients and regulates leaf temperature, and the other is to control CO_2_ entry into the leaf. Under drought conditions, one of the first response of plants is a reduction in the stomatal aperture, and when the stress event continues, both changes in leaf photochemistry and/or carbon metabolism are also impaired, therefore, negatively affecting photosynthesis (Dias et al., 2014; Vasques et al., 2016; Araújo et al., 2019). The reduction of the stomata aperture width (stomatal closure) prevents the loss of water to the atmosphere and this protection mechanism is considered an adaptation response of plants to the onset of drought conditions (Saradadevi et al., 2017). Besides, CO_2_ diffusion through the leaf mesophyll cells (mesophyll conductance) is also affected, mostly due to changes in leaf biochemistry, membrane permeability (aquaporin activity), and leaf shrinkage (modification of the intercellular spaces and structures; Chaves et al., 2009). Following stomatal closure, the low CO_2_ availability in the leaf intercellular spaces can impair the biochemical reactions. This result in a reduction or de-activation of the enzyme ribulose 1,5-bisphosphate carboxylase/oxygenase (RuBisCO), or other Calvin cycle enzymes, such as ribulose-5-phosphate kinase, or a decrease in the substrate of RuBisCO (regeneration of RuBP; Dias and Brüggemann, 2011; Galmés et al., 2011; Perdomo et al., 2017; Wang et al., 2018a). RuBisCO amount, activity, and alterations at the transcript level were reported even under mild water deficit (Zhang et al., 2013). Also, the ROS generated under stress conditions in the chloroplast is reported to damage ATP synthase and decrease ATP production, which consequently leads to lower ribulose-1,5-bisphosphate (RuBP) regeneration (Pinheiro and Chaves, 2011).

When plants are exposed to high light intensities and the inhibition of biochemical reactions by drought conditions precedes the inhibition of light-dependent reactions of photosynthesis, the rate of reducing power production can overcome the rate of its use in the Calvin cycle. Under this condition, the excess of light energy has to be dissipated to avoid overexcitation, and consequential damages in the photosynthetic machinery, particularly in photosystem II (PSII). Plants possess a range of protective mechanisms against the excess of light energy that decrease the probability of damage in PS reaction centers. These mechanisms include dissipation of absorbed light energy as thermal energy (non-photochemical quenching, involving the xanthophyll and the lutein cycle), pseudocyclic electron flow coupled to ROS scavenging systems, PSI cyclic electron flow, increased photorespiration, changes in leaf angle, and chloroplast avoidance movement (Pinheiro and Chaves, 2011; Wang et al., 2018b). Additionally, the protection of anthocyanins over chlorophylls also contributes to plant photoprotection (Gould et al., 2002). Anthocyanins function as light screening pigments, filtering the extra photons that would be damaging if absorbed by chlorophylls (Nichelmann and Bilger, 2017; Gould et al., 2018). Moreover, the anthocyanins located in the leaf epiderm seem more efficient to photoprotect the subjacent mesophyll from photoinhibition (Lo Piccolo et al., 2018).

Under salinity stress, stomata closure and consequent reduction of the intercellular CO_2_ concentration are the main causes of photosynthesis impairment. This decrease leads to reductions in the carbohydrate pool and protein concentration. Salt stress also affects other photosynthetic components such as the efficiency of RuBisCO for carbon fixation, and the enzymes related to chlorophylls and carotenoid biosynthesis (Demetriou et al., 2007; Chaves et al., 2009). The light reactions of photosynthesis were reported to be very susceptible to salt stress. Photosynthesis inhibition by salinity seems to be partially associated with the PSII complex. Salt stress reduces the PSII activity and the quantum yield of PSII electron transport and affects the photosynthetic pigment-protein complexes (Demetriou et al., 2007). Changes in these photosynthetic parameters depend on the intensity and duration of the stress, but plant species is also an important factor. For instance, both *Sorghum bicolor* salt-sensitive and -tolerant genotypes showed a reduction in net CO_2_ assimilation rate and PSII efficiency (F_v_/F_m_ and *Φ*_PSII_), but in the sensitive genotype, these effects were stronger (Sui et al., 2015). In several *O. sativa* cultivars with different salt-tolerances, the *Φ*_PSII_ and net CO_2_ assimilation rate were also negatively affected, but the F_v_/F_m_ was maintained under optimal values (0.75–0.80; Yang et al., 2020).

### Chlorophyll Content

Photosynthetic pigments are essential for light harvesting and, hence, for photosynthesis and plant growth. The decrease in chlorophyll content under drought or salinity stress, caused by pigment photooxidation and chlorophyll degradation, is considered a symptom of oxidative stress. Drought and salinity have been found to induce a reduction in chlorophyll contents in leaves of various crops, such as *Carthamus tinctorius* (Siddiqi et al., 2009), *Phaseolus vulgaris* (Beinsan et al., 2003), *Vigna subterranean* (Taffouo et al., 2010) and *P. vulgaris* (Taïbi et al., 2016), indicating the process of photooxidation (Rahdari et al., 2012). The reduced chlorophyll contents under drought or salinity stress may trigger the inactivation of photosynthesis. Moreover, drought or salinity induced reduction in chlorophyll content is contributed to excessive chloroplast swelling, loss of chloroplast membranes, the appearance/development of intracellular lipid droplet, and distortion of lamellae vesiculation (Kaiser et al., 1981). Low photosynthetic pigment content may hamper photosynthetic potential and thus primary plant production.

### Phytohormonal Regulation

Plant stress response mechanisms are highly complex comprising the integration of several pathways. The perception of a stress event starts signal transduction cascades that interact with pathways traduced by phytohormones (Harrison, 2012). Endogenous phytohormones are not only important growth regulators but also play a pivotal role in plant adaptation to drought and salt stress by modulating plant physiology and molecular responses (Fahad et al., 2015; Wani et al., 2016). Water deficit and salt stress may alter the biosynthesis, accumulation, and distribution of several phytohormones, including ABA, jasmonic acid (JA), salicylic acid (SA), indole-3-acetic acid (IAA), gibberellins (GA), and cytokinins (CK), therefore, promoting specific protective mechanisms (Eyidogan et al., 2012).

Abscisic acid is one of the most important phytohormones and the major stress-responsive hormone produced after signal perception by drought and salt stress (Fahad et al., 2015; Ullah et al., 2018). Stress perception triggers the synthesis of ABA predominantly in roots (however ABA can also be synthesized in leaf cells and translocated around the plant) and acts in the regulation of the stomatal aperture, channel activities, and in the expression of ABA-responsive genes (Ullah et al., 2018). In leaves, ABA increases after drought or salt stress and regulates stomatal aperture (promote stomatal closure), consequently helping plants to control the water status and preventing dehydration. For instance, as a result of drought, leaf mesophyll cells come to be dehydrated, causing a release of ABA. The amount of ABA is then stored in the chloroplasts of guard cells. The increased concentration of ABA triggers the efflux of potassium (K) and calcium from the guard cells, leading to stomatal closure with water loss in the guard cells. The shortage of water causes discoloration, a decrease in the rate of photosynthesis in plants, and an increase in leaf trichomes and stomata on the leaf surface. Besides, ABA increases the production of ROS (especially H_2_O_2_) in guard cells to decrease the stomatal aperture (stomata closure; Golldack et al., 2014; Mittler and Blumwald, 2015). Furthermore, ABA is also involved in root architectural modifications by promoting root elongation to reach deep water in the soil during water deficit conditions and can upregulate the synthesis of osmoprotectants (e.g., proline), antioxidant enzymes, and the expression of several drought and salt stress-responsive genes and proteins (e.g., late embryogenesis abundant proteins and dehydrins; Comas et al., 2013; Fahad et al., 2015; Mittler and Blumwald, 2015; Wani et al., 2016; Ullah et al., 2018). Some works also demonstrated that ABA induces modification of primary lipid metabolism, contributing to stress adaptive reorganization of membranes (e.g., change the monogalactosyldiacylglycerol and digalactosyldiacylglycerol contents in the chloroplast envelope and thylakoid membranes) and to the maintenance of cellular energy supply under drought and salt stress (Golldack et al., 2014).

Jasmonic acid and its derivatives, the jasmonates, besides being involved in reproductive processes, root growth, and fruit ripening, also play a crucial role in drought and salt stress response regulation in plants (Fahad et al., 2015; Wani et al., 2016). Biosynthesis of JA occurs usually in leaves, particularly in chloroplasts and peroxisomes (Cheong and Choi, 2003), despite some reports also refer to the roots as a synthesis organ of this hormone (Fahad et al., 2015). Similar to ABA, JA induces drought and salinity tolerance in plants in various ways, such as inducing stomatal closure, scavenging of ROS, and promoting root development. Walia et al. (2007) proposed that JA induced salinity tolerance by regulating arginine decarboxylase, RuBisCO activase, and apoplastic invertase.

Salicylic acid is a naturally occurring phenolic compound, which is usually involved in the regulation of pathogen-associated protein expression (Miura and Tada, 2014). Besides, several works demonstrated that SA also has an important role in plant defense against drought and salt stresses (Khodary, 2004; Fahad and Bano, 2012; Miura et al., 2013). Low concentrations of SA enhance the antioxidant capacity of plants, but high concentrations can cause cell death or even some susceptibility to abiotic stresses (Jumali et al., 2011). SA activates the expression of genes involved in the biosynthesis of secondary metabolites, chaperones, and heat shock proteins (Miura and Tada, 2014). SA biosynthesis takes place mostly in the chloroplasts being then transported to other parts of the plant.

Indole-3-acetic acid is widely acknowledged for its implications in plant growth and development (e.g., cell elongation, vascular tissue development, and apical dominance; Fahad et al., 2015). Also, during water deficit and salt stress, this phytohormone seems to coordinate growth (Eyidogan et al., 2012; Iqbal et al., 2014) and induce the expression of genes related to root meristem initiation, promoting root branching and increasing plant stress tolerance (Wolters and Jürgens, 2009).

### Osmotic Adjustment

Osmotic adjustment (OA), defined as the lowering osmotic potential as a result of net solute accumulation in response to water stress, is well known to have a significant role in plant adaptation to dehydration *via* the maintenance of turgor pressure, relative water content, stomatal conductance, and specific cellular functions (Martínez et al., 2004). The accumulation of compatible solutes in plants is thought to benefit stressed cells either by acting as cytoplasmic osmolytes to facilitate water uptake and retention, or by protecting macromolecules (e.g., proteins, chloroplast, and membranes) and their structure from stress-induced damage (Martínez et al., 2004). It is well known that plant growth is inhibited by drought before photosynthesis, causing the release of sugars toward OA. Moreover, OA can sustain photosynthesis by maintaining turgor as stress develops. Therefore, OA inhibits plant growth and protects photosynthesis simultaneously. Moreover, as one of the most important physiological parameters of plant adaptation to drought, cell wall elasticity (CWE) plays a key role in turgor regulation. The increases in CWE have been found in several plant species when they respond to drought, which may contribute to maintaining cell turgor or symplast volume (Marshall and Dumbroff, 1999). Under osmotic stress, plants can avoid reduced water potential and sustain turgor by decreasing their turgor-loss volume *via* shrinkage associated with cell wall elastic adjustment (Marshall et al., 1999). Cell shrinkage/contraction (a reduction in cell size) has been considered as the main character involved in plant resistance to drought stress. For instance, Alves and Setter (2004) have found a reduction in cell size and osmolyte accumulation in *Manihot esculenta* grown under drought stress.

Plants generally respond to salinity stress using different mechanisms, depending on the severity and duration of the stress. Soil salinity initially hinders plant growth in the form of osmotic stress (hyperosmotic stress) followed by ion toxicity (hyperionic stress; Munns and Tester, 2008). During the initial phase of salinity stress, root water absorption capacity diminishes, and the leaf water loss is augmented because of salt-induced osmotic stress in plants. This hyperosmotic stress results in various physiological alterations in plants, such as membrane disruption, nutrient imbalance, ROS detoxification, antioxidant enzyme activity, and photosynthetic activity. The latter phase is a hyperionic stress due to Na^+^ and Cl^−^ uptake and their subsequent accumulation in leaves, leading to nutritional imbalance (e.g., inhibition of K^+^ uptake) and physiological disorder (e.g., significant alterations in the metabolism; Munns and Tester, 2008). It is well known that the accumulations and functions of compatible solutes, such as soluble sugars, proline, glycine betaine, and sugar alcohols, play an essential role in OA. The distribution of photoassimilates between source and sink tissues can contribute to accumulating these solutes (Hare et al., 1998). However, the role of these solutes in OA under salinity stress is still under debate. In many plant species, the absolute osmolyte concentrations are doubtful to mediate OA. However, these solutes have beneficial potential to buffer cellular redox potential, protect the cellular structure, and adaptively modify carbon allocation and sugar metabolism under salt stress. For instance, Suwa et al. (2008) observed a decrease in carbon allocation toward the roots of *Lycopersicon esculentum*, even before the salt symptoms of reduced photosynthesis. As a result of such changes in carbon distribution, the accumulation of sugar in mature leaves is higher under salt stress. The changes in sugar homeostasis and metabolism have been found in plants under salt stress (Peng et al., 2016). These changes are associated with cell wall modification by either stiffening cell walls to reduce the salt entry or enhancing cell wall elasticity to maintain cell turgor (Gall et al., 2015). However, sugar transport and homeostasis as well as cell wall viscoelastic properties are tightly regulated during plant development. Therefore, alterations in plant susceptibility to salt stress are possible, and they are determined by carbon status (Wingler, 2017).

### Source/Sink Dynamics

In general, carbon assimilation is performed by source organs (exporters of photoassimilates, e.g., fully developed leaves), which converts it into glucose and other sugars and then exports them to sink organs (importers of fixed carbon, e.g., roots, stems, fruits, and seeds) for plant organ growth (Yu et al., 2015). During plant growth, communication between source organs and sink organs plays an important role in carbohydrate assimilation and partitioning/allocation that are strictly connected to photosynthesis. Carbohydrates, the end product of photosynthesis, after being exported from the leaves to non-photosynthetic tissues provide the substrate for growth and cell maintenance. Sugar transporters are required for carbohydrate allocation to long-distance at the plant level and a short distance in sugar partitioning at the cellular level. Source-to-sink transport of sugar has been considered as the main factor affecting plant growth and it depends on the proficient and controlled distribution of sugars across plant organs *via* the phloem. Nevertheless, sugar transport *via* the phloem can be influenced by various environmental stresses at three diffident levels (namely the source, the sink, and the path between source and sink) that may change source/sink relationships (Lemoine et al., 2013).

Under drought or salt conditions, the reduction of global photosynthetic productivity can be aggravated, therefore, impacting the carbon flow to different sink organs (Courtois et al., 2000; Lebon et al., 2006; Penella et al., 2016). The impact of drought and salinity on sugar metabolism and phloem loading has been widely investigated (Burke, 2007; Hummel et al., 2010; Lemoine et al., 2013). In sucrose-translocating species, carbohydrate levels in leaves and source-to-sink translocation patterns are generally altered by drought or salinity stress as the result of reduced photosynthesis (Pattanagul and Thitisaksakul, 2008). Sucrose and hexose accumulations contribute significantly to osmotic modulation to sustain metabolic activity in source leaves. However, the concentration of sugars may increase in leaves due to reduced demand as a consequence of growth limitation.

Under drought stress, transcript abundance of several genes encoding gluconeogenic enzymes including fructose biphosphate aldolase (Cramer et al., 2007), hexokinase in phosphorylation of soluble sugars (Whittaker et al., 2001), and galactinol synthase in raffinose family oligosaccharide biosynthesis (Taji et al., 2002) increased in source leaves. Moreover, drought may also cause changes in the concentrations of organic nutrients (e.g., sugars and amino acids). In sink organs, the presence of drought stress can promote sucrose biosynthesis rather than starch biosynthesis by inducing sucrose-phosphate synthase and hindering ADP glucose pyrophosphorylase (Geigenberger et al., 1997). At different development stages, drought may induce senescence and increase reserve mobilization, which is considered as a component of basic strategies for plant development and stress alleviation (Chandlee, 2001). In general, drought can cause higher sugar content in the cytosol to lower osmotic potential, therefore, maintaining cell turgor (Razavi et al., 2011). This would eventually diminish photosynthetic actives and thus accelerate senescence in leaves. The drought-induced increase in sugar levels in plants may be due to the attempt of plants to adjust their metabolism to maintain the osmotic homeostasis (Giné-Bordonaba and Terry, 2016). Sugar concentrations may affect leaf development *via* senescence as direct causal signals, and as substrates for C mobilization and reallocation to help host plants mitigate the negative effects of drought (Cramer et al., 2007).

Salinity stress generally results from irrigation with poor quality water and it shares many similar features with drought stress, particularly in the early stress response, as the primary effect induced by both stresses inhibits the absorption of water through the root system due to the osmotic effect (Navarro et al., 2008). However, the long-term plant responses to both stresses may behave differently, because sodium toxicity can add to the initial stress due to its transport within plant tissues *via* the transpiration stream. The K^+^ channels are involved in the Na^+^ recirculation from leaf phloem to roots for exertion, thus diminishing the amount of Na^+^ in leaves (Berthomieu et al., 2003). There is a lack of information about the positive impact of salinity on sucrose translocation into the phloem. As discussed above, salinity stress can adversely affect photosynthesis, thus leading to plant growth impairment, especially leaves (Suwa et al., 2006). In the study of Lohaus et al. (2000), salinity did not change sucrose concentration in the phloem of *Z. mays*, whereas increased amino acid and Na^+^ concentration in the sieve tube sap. The increased root/shoot ratio was probably attributed to the fact that the higher amount of amino acids was transported to the roots. Dissimilarly, Suwa et al. (2008) found that salinity stress inhibited phloem sucrose uptake and translocation, causing the insufficient distribution of sucrose to the roots. Some salt-tolerant plant species do not tolerate drought stress and vice versa (Kefu et al., 2003). The specific mechanisms that plants use for salt stress alleviation are either by minimizing the entry of salt ions into plant tissues or reducing the salt concentrations in the cell cytoplasm. In general, halophytes (plants that tolerate high concentration of salt) can exclude most of the Na^+^ and Cl^−^ into the soil solution and compartmentalize salts in cell vacuoles, therefore, achieving salt tolerance, whereas most of the glycophytes (plants that tolerate relatively low concentration of salt) have a low potential to exclude salt and, therefore, accumulate toxic ions in the leaves with the transpiration flow (Munns, 2002).

Besides, polyols are thought to be the major osmotically active and antioxidant molecules for plants to cope with stress (Stoop et al., 1996). When plants are exposed to salinity stress, the polyol concentration usually increases in different plant tissues. This is probably the reason why salinity resistance/tolerance is commonly found in polyol synthesizing plants. Furthermore, the increased polyol synthesis together with an increased expression of genes encoding polyol transporters was observed in the phloem of *Plantago major* (Pommerrenig et al., 2007), *Apium graveolens* (Landouar-Arsivaud et al., 2011), and *Olea europaea* (Conde et al., 2011), suggesting that long-distance polyol transport is induced/enhanced by salinity stress. The translocation of polyols from shoots to roots may positively affect root metabolism and water potential.

### Antioxidants

Both drought and salinity can induce the formation of ROS such as superoxide, singlet oxygen, hydrogen peroxide, and hydroxyl radicals within plant cells, and their overaccumulation results in oxidative damage of membrane lipids, proteins, DNA, and nucleic acids in plants (Gill and Tuteja, 2010). To scavenge high levels of ROS, the efficient non-enzymatic (e.g., ascorbate, flavonoids, glutathione, tocopherols, and phenolics) and enzymatic [e.g., catalase (CAT), peroxidase (POD), ascorbate peroxidase (APX), and superoxide dismutase (SOD)] antioxidant defenses system is involved. The upregulation of antioxidants has been found in drought or salinity tolerant cultivars of various crops, such as *Calendula officinalis*, *Solanum lycopersicum*, *Jatropha curcas*, and *Z. mays* (Chaparzadeh et al., 2004; Mittova et al., 2004; Gao et al., 2008; Anjum et al., 2017), implying the great potential of antioxidants to ameliorate drought/salt-induced oxidative stress. Maintaining a high level of antioxidative enzyme activities contributes greatly to drought or salt stress alleviation by enhancing the capacity of host plants against oxidative damage. Therefore, the ability of antioxidant enzymes to scavenge ROS and diminish the damaging impacts are closely related to plant drought or salinity stress resistance. Besides, drought or salinity can adversely affect various subcellular compartments (e.g., vacuole, cytoplasm, and nucleus), cell organs, and whole plant level (Rahdari et al., 2012), consequently affecting plant biomass and health. Thus, the alleviation of drought and salinity stresses is important to achieve a healthier food growing environment.

## Metabolic Responses to Drought and Salinity Stress

Stress conditions strongly affect plant metabolism, resulting in deep modifications in metabolites biosynthesis, transport, and storage (Fraire-Velázquez and Balderas-Hernández, 2013; Di Ferdinando et al., 2014). A quick metabolic response at the beginning of the stress event helps the plant to restore its performance faster and is crucial to stress acclimation and further plant survival (Fraire-Velázquez and Balderas-Hernández, 2013).

Metabolomic studies can help to identify key stress metabolite that could be useful as indicators of the adaptability of plants/species to drought or salinity, to detect the adjustments of groups of compounds involved in mediating the stress tolerance, and to investigate the flexibility of a species to rearrange principal metabolic pathways (such as carbon and nitrogen metabolism) to tolerate and/or adapt to various stresses (Jorge and António, 2018). Within the metabolic changes, both primary (e.g., osmolytes) and secondary (e.g., defense compounds) metabolic adjustment have been reported. Primary metabolites are essential for plant growth and development, being more conserved in their abundance within species, while secondary metabolites (besides it is not necessary for survival) play a role in the interaction of the plants with their environment and, therefore, differ more across the species (Jorge and António, 2018).

Under drought conditions, the adjustment of photosynthesis and osmoregulation is one of the earliest plant strategies (Slama et al., 2015). Metabolomic studies identified the accumulation of osmolytes, such as some carbohydrates (e.g., glucose, sucrose, trehalose, and raffinose), polyols (e.g., mannitol and sorbitol), amino acids (e.g., proline, betaine, valine, leucine, and isoleucine), quaternary ammonium compounds (e.g., glycine betaine, *b*-alanine betaine, and proline betaine), and polyamines (e.g., putrescine, spermidine, and spermine), which have an important role in the decrease of the osmotic potential and maintenance of cell turgor pressure (as the cell uptakes water), and contribute to the stabilization of membranes, enzymes, and proteins (Jorge and António, 2018; Sharma et al., 2019). Also, the accumulation of osmolytes helps to control ROS levels, provides energy to cope with stress, contributes to repair processes, and supports further growth (Silva et al., 2018; Fàbregas and Fernie, 2019). Other metabolites related to the tricarboxylic acid (TCA) cycle were identified in several metabolomics studies, but these compounds respond more heterogeneously to drought. For example, several reports revealed contrasting results, describing increases of TCA acids (e.g., isocitrate, oxoglutarate, succinate, fumarate, and malate) in *Arabidopsis* (Fàbregas et al., 2018), while in rice, showed decreased levels (Todaka et al., 2017). TCA cycle metabolites provide the precursors for energy generation and are also an integral part of the oxidative defense machinery (Mailloux et al., 2007). The overproduction of ROS is one of the primary consequences of the impairment of photosynthesis under drought conditions, and some TCA cycle compounds, such as *α*-ketoglutarate, participate in the detoxification of ROS (Fàbregas and Fernie, 2019). Besides, drought also upregulates the biosynthesis of other antioxidant compounds, leading to the accumulation of ascorbate, glutathione, and several polyphenols (Das and Roychoudhury, 2014). Among plant defense metabolites, the polyphenols with ROS scavenger capacity (e.g., anthocyanins, 4-coumaric acid, caffeic acid, ferulic acid, cis-resveratrol-3-*O*-glucoside, trans-resveratrol-3-*O*-glucoside, catechin, epicatechin, caftaric acid, kaempferol and kaempferol-3-*O*-glucoside, cyanidin-3-*O*-glucoside, rutin, quercetin and quercetin-3-*O*-glucoside, luteolin and luteolin-7-*O*-glycoside, and apigenin and chlorogenic acid) were the most responsive to drought (Di Ferdinando et al., 2014; Sharma et al., 2019). For instance, the rapid accumulation of the anthocyanins and flavones may act as vacuole ROS scavengers in response to drought (Fàbregas and Fernie, 2019). Also, other flavonoids like kaempferol and quercetin were enhanced by drought, participating efficiently in the detoxification of H_2_O_2_ molecules (Sharma et al., 2019). Lipophilic compounds are also sensitive to drought. The compounds involved in stress detoxification and tolerance, such as 2-naphthalene methanol, heneicosane, caryophyllene oxide, heptadecanal, tetratetracontane, heptatriacotanol, tetracosane, 1-heptacosanol, phytol, *n*-nonadecanol-1, *n*-pentadecanol, octacosyl acetate, octadecanoic acid, and hexatriacontane, increased significantly in response to water deficit (Soliman et al., 2019).

Under salinity, osmotic stress affects cell functions, leading to cell damages and a slowdown of plant growth. Sodium accumulation in cells induces secondary stress that affects negatively some principal processes, such as protein biosynthesis, photosynthesis as well as potassium ion absorption (Deinlein et al., 2014). Metabolite responses to salt stress have much in common with drought, evidencing an overlapping of biochemical pathways and similar metabolite adjustments. For instance, the accumulation of osmolytes is also an important adaptive mechanism to salinity, enabling cell turgor maintenance, and reposition of water status, membrane stabilization, and ROS increase control (Kao, 2015). Several metabolomic studies revealed that salt stress induces the accumulation of few primary metabolites, such as proline or primary products in the N metabolism (such as allantoin, urea, and glutamine; Slama et al., 2015). Also, some carbohydrates, such as sucrose, tagatose, psicose, glucoheptose, idose, allose, talose, lactulose, and cellobiose, and the sugar alcohol (inositol) respond with a sharp increase under salinity. Salt stress induces an over-accumulation of ROS (e.g., superoxide anions, hydroxyl ions, and hydrogen peroxide), resulting in damages in membranes and macromolecules (Sharma et al., 2019). The phenolic compounds have an important role in acting as powerful antioxidants that help in scavenging ROS. Metabolomic studies revealed that the phenylpropanoid biosynthetic pathway is one of the most stimulated by salinity. The stimulation of this pathway results in the increased production of various phenolic compounds, such as hydroxybenzoic acids (e.g., gallic acid, vanillic acid, syringic acid, p-hydroxybenzoic acid, and ellagic acid), hydroxycinnamic acids (e.g., caffeic acid, chlorogenic acid, *p*-coumaric acid, *m*-coumaric acid, ferulic acid, sinapic acid, and trans-cinnamic acid), and flavonoids (e.g., quercetin and iso-quercetin, rutin, luteolin and luteolin-7-*O*-glycoside, apigenin, kaempferol, and luteolin; Sharma et al., 2019). Accumulation of lipophilic compounds, such as α-tocopherol implicated in antioxidant responses (e.g., oxygen free radical, lipid peroxyl radicals, and ^1^O_2_ – scavenging capacity) in different species were found to activate the resistance to salinity (Malik et al., 2013). Carotenoids, another group of lipophilic antioxidants that can remove several types of ROS, were found to accumulate in sugar cane under salt stress (Malik et al., 2013).

In general, drought and salinity alter several metabolic pathways. However, some of them are not well studied and the potential of these metabolic changes is not completely and scientifically explored. Therefore, it is important to understand better, for instance, how controlled stress situations (e.g., low or moderate water deficit) shifts metabolic pathways resulting in the enhancement of metabolites that, besides protecting plants, can also alter its nutritional value. For instance, the increase of some metabolites, such as *β*-carotene in *Brassica chinensis* var. *parachinensis*, vitamin C in *S. lycopersicum*, polyphenols in *Fagopyrum esculentum*, stevioside in *Stevia rebaudiana* leaves, allicin in *Allium sativum*, and rosmarinic acid in *Salvia miltiorrhiza* leaves, in response to water deficit (Sarker and Oba, 2018; Isah, 2019) enhance the quality of these species/fruits. Moreover, the implications of these metabolic changes in plant growth and productivity must also be taken into consideration.

## Plant-Microbe Interactions Confer Abiotic Stress Tolerance

Plant-microbe interactions are considered as an essential determinant of ecosystem processes (Cheng et al., 2019). Plants are living intimately with the microbial communities in the root system (Friesen et al., 2011). In general, the roots shape the niche or environment where the microbial communities establish and survive, while the plant-associated microbes, especially plant growth-promoting microorganisms (PGPM) can affect the growth, nutritional status, development, and fitness of the host plants (Pascale et al., 2020).

### Role of PGPM in Abiotic Stress

Plant growth-promoting microorganisms including plant growth-promoting bacteria (PGPB), rhizobia, and arbuscular mycorrhizal fungi (AMF) are defined as microbes inhabiting around/in free-living soils, rhizosphere/rhizoplane (e.g., rhizobacteria and ectomycorrhizal fungi), or tissue interior (e.g., endophytic bacteria, endomycorrhizal fungi, and AMF) that are beneficial for plants (Ma et al., 2011). The role of PGPM in plant growth, nutrient acquisition, and biocontrol activity has been well established. Despite the difference between these types of microbes, these PGPM strains can colonize the rhizosphere soils or endo-rhizosphere of plants and they can protect plants from both abiotic (e.g., drought, salinity, and extreme temperature) and biotic stresses (e.g., phytopathogens) and enhance plant establishment and growth *via* the same plant growth-promoting mechanisms that involve direct and indirect mechanisms (Ma et al., 2019b). In general, the direct mechanisms include the facilitation of nutrient acquisition (e.g., nitrogen fixation, siderophore sequestration, and potassium and phosphate solubilization; Kohler et al., 2009), synthesis of phytohormones (e.g., auxin, cytokinin, ABA, and GA; Ma et al., 2019a), exopolysaccharides (EPS; Naseem et al., 2018), and volatile or non-volatile compounds, as well as induction of ACC deaminase (Forni et al., 2017). Indirect mechanisms include biological control against phytopathogens (e.g., bacteria, fungi, and nematodes) through the synthesis of allelochemicals (e.g., antibiotics and antifungal metabolites) or induced systemic resistance (e.g., reinforcement of plant cell wall, production of antimicrobial substances, and the synthesis of pathogen-related proteins; Ma et al., 2011). As the abiotic stresses (drought and salinity) have adverse impacts on crop yields, the potential role of PGPM in improving plant performance makes it important to elucidate the responses of plant-associated microbes to environmental change (Compant et al., 2010).

### Drought and Salinity Stress Microbial Ecology

Drought or salinity could significantly reduce plant yields, cause land degradation, and influence plant-microbe interactions (Munns, 2002; Lesk et al., 2016). Recently, Santos-Medellín et al. (2017) explored the responses of rice root-associated microbiomes to drought stress and they found major compositional changes in the rhizosphere and endosphere communities, particularly changes in the relative abundance of taxonomically diverse bacteria in response to drought. Drought-resistant PGPM might potentially benefit the host plants and improve their adaption to various abiotic stresses, due to their contribution to plant tolerance to drought and their ability to protect plants from infection by pathogens (Ma et al., 2016, 2017; Tiepo et al., 2018; Eke et al., 2019; Halo et al., 2020). The isolation, characterization, and identification of microbes possessing the ability to promote plant tolerance to drought might be used to alleviate crop losses under adverse climate change conditions. Likewise, Xu et al. (2018) demonstrated that drought greatly diminished bacteria community diversity in the rhizosphere and root endosphere, and increased the abundance of *Actinobacteria* and *Firmicutes*, which were most pronounced in the root endosphere. Besides, drought stress resulted in a shift in the metabolites secreted by the roots of host plants. The findings suggest that there are molecular dialogs/interactions between host plants and their associated microbes for reshaping rhizosphere biota to cope with/adapt to drought stress. Unraveling the molecular dialogs may advance fundamental knowledge of employing beneficial microbes to improve plant stress tolerance. However, there is a lack of information about whether and how the drought-enriched metabolites deploy/drive rhizosphere microbial composition.

So far, the root microbial structure, composition, and function under natural and agricultural environments have been extensively explored. However, there has been no coordinated effort to dissect the impacts of extreme environments (e.g., drought, salinity, and heat) on microbial community composition for helping understand the mechanisms of reshaping plant-microbe interactions under changing climatic conditions (Busby et al., 2017). Also, the identification of rhizosphere microorganisms that thrive under different adverse environmental conditions can result in the discovery of beneficial symbiosis, as the microbial traits that confer stress tolerance may be beneficial to the plant hosts (Rodriguez et al., 2008). By the end of this century, the frequency of drought is expected to increase, and this trend may gradually change the underground characteristics of the affected agricultural ecosystems. Although drought may reconstruct the soil bacterial diversity (Barnard et al., 2013; Bouskill et al., 2013), little is known about the impact of drought on microbial communities in the rhizosphere of various plant species. These microbial communities might be directly affected by drought stress and/or indirectly by host-mediated processes since drought triggers a series of plant molecular, physiological, and developmental responses (Xu et al., 2010; Gray and Brady, 2016).

Salinity stress is known to influence both bacterial and fungal diversity in different manners, by driving the soil nutrient cycle in various land ecosystems. In general, bacterial diversity decreases with salinity, whereas the response of fungi to this stress is more complex. Bacterial and fungal community structure depends on the levels of salinity (Thiem et al., 2018). Zhang et al. (2019a) reported that the increasing soil salinity decreased the relative abundances of soil bacterial, fungal, and arbuscular mycorrhizal communities and, thereafter, affected their function (e.g., organic matter decomposition and lignin degradation) in saline coastal ecosystems.

### Mechanisms of PGPM Mediated Drought and Salinity Tolerance

**Figure 3:**
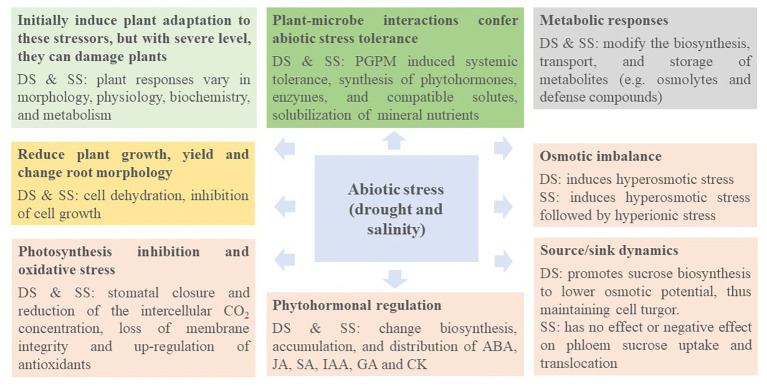
Mechanisms underlying abiotic stress response and adaptation. DS, drought stress; SS, salinity stress; PGPM, plant growth-promoting microorganisms; ABA, abscisic acid; JA, jasmonates; SA, salicylates; IAA, indole-3-acetic acid; GA, gibberellins; CK, cytokinins.

Several strategies have been explored to alleviate the toxic/detrimental effects induced by abiotic stress on plant growth and development, including plant genetic engineering, and recently the application of PGPM (Dimkpa et al., 2009; Ma et al., 2016, 2017, 2019a,b). Although Yang et al. (2009) and Dodd and Perez-Alfocea (2012) have extensively reviewed the use of PGPB in plants as elicitors of tolerance to abiotic stresses, we attempt to shed light on the underlying mechanisms used in various PGPM species to assist crops to cope with drought and salinity stresses. As the basic mechanisms behind drought and salinity stresses are similar, they are discussed together under common headings (Figure 3).

#### Microbial Induction of Systemic Resistance

Plant growth-promoting microorganisms induced systemic tolerance (IST) may lead to physical and chemicals changes in plants, thus contributing to plant tolerance to abiotic and biotic stresses. Many researchers have advocated that crops inoculated PGPM persuade morphological and biochemical changes resulting in increased drought tolerance by eliciting IST (Yang et al., 2009; Ma et al., 2016, 2017; Vurukonda et al., 2016). Facultative endosymbionts can confer adaptive advantages to their hosts, such as digestion, fecundity, and resistance to abiotic (e.g., drought, salinity, and heat) or biotic stresses (Guo et al., 2017). IST is accompanied by PGPM by allopathic compound production, competition for ecotype and nutrient. Allelochemicals comprising antibiotics, lytic enzymes, and siderophores, act effectively against pathogens and suppress their growth (Jain et al., 2013). Frago et al. (2017) demonstrated that *Hamiltonella defensa*, an endosymbiont of aphids and other sap-feeding insects, protected plants against parasitoids and diminished the emission of plant defensive volatiles following aphid attack.

#### Nutrient Acquisition and Ion Homeostasis

Plant growth-promoting microorganisms have a great capacity to convert nutritional elements from unavailable to available form through biological processes (Ma et al., 2011). Their performances are greatly influenced by various environmental factors, such as soil type and properties, metal contamination, and abiotic stress (e.g., drought and salinity). Egamberdiyeva (2007) found that three PGPB isolates *Pseudomona alcaligenes* PsA15, *Bacillus polymyxa* BcP26, and *Mycobacterium phlei* MbP18 could tolerate high temperatures and salt concentrations and have stimulatory effects on nitrogen, phosphorus, and potassium uptake of *Z. mays* in nutrient-deficient calcisol soil, thus enhancing the ability of the plant to survive in arid and saline soils. Under drought stress, mycorrhizal association with the plants can improve nutrient accumulation *via* the establishment of extensive hyphal networks and glomalin secretion that facilitate water and nutrient uptake (Bahadur et al., 2019).

It is well known that salinity inhibits plant growth owning to increased Na^+^ concentration and low K^+^/Na^+^ ratio in plants. A number of studies have demonstrated that PGPM inoculation avoided the over-accumulation of Na^+^ and maintained the ion homeostasis under salinity stress (Bharti et al., 2014; Tewari and Arora, 2014). Kohler et al. (2009) examined the effects of inoculation of plant growth-promoting rhizobacterium *Pseudomonas mendocina*, alone or in combination with AMF *Glomus intraradices* or *Glomus mosseae* on the growth, nutrient accumulation, and physiological parameters of *Lactuca sativa* exposed to salt stress. The results showed that *L. sativa* inoculated with *P. mendocina* reduced plant Na^+^ uptake and increased K^+^ uptake, resulting in a higher K^+^/Na^+^ ratio, as well as greater shoot biomass, compared to the control treatments, suggesting that the application of PGPB could be used to alleviate salinity stress in plants that are sensitive to salt. The volatile organic compounds synthesized from *Bacillus subtilis* decreased transcriptional expression of a high-affinity K^+^ transporter (HKT1) in *Arabidopsis* roots whereas it was upregulated in shoots, thus reducing Na^+^ uptake by roots and Na^+^ expulsion from shoots (Zhang et al., 2008).

#### Phytohormonal Modulations

Plant growth-promoting bacteria can produce phytohormones such as IAA, GA, ABA, etc., which stimulate plant cell growth and division to become tolerant/resistant to environmental stresses (Ma et al., 2019a). Synthesis of IAA and GA by PGPB caused an increase in plant growth (e.g., root length, root surface area, and the number of root tips) and nutrient uptake, thus ameliorating plant health under drought and salinity stress (Egamberdieva and Kucharova, 2009; Ma et al., 2016, 2017). Several studies have found that PGPB can improve the growth of various plant species (such as *S. lycopersicum*, *Solanum pimpinellifolium*, *Capsicum annuum*, *B. napus*, *Helianthus annuus*, *P. vulgaris*, *T. aestivum*, and *L. sativa*) under drought or saline conditions (Yildirim and Taylor, 2005; Barassi et al., 2006; Hussain et al., 2014; Ma et al., 2016, 2017, 2019a; Khan et al., 2017).

The abscisic acid produced by PGPB can also contribute to microbe-induced drought or salinity tolerance. PGPB *Phyllobacterium brassicacearum* STM196 improved osmotic stress tolerance in *Arabidopsis thaliana* by increasing ABA content, thus inducing a reduction in leaf transpiration (Bresson et al., 2013). Likewise, inoculation of cytokinin producing PGPB *B. subtilis* enhanced the ABA content in the shoots of *Platycladus orientalis* seedlings and the stomatal conductance, consequently conferring drought stress tolerance (Liu et al., 2013). In terms of salinity stress, Naz et al. (2009) found that the bacterial strains isolated from Khewra exhibited salt tolerance and were able to produce IAA, GA, trans-zeatin riboside, and ABA. Moreover, the inoculation of these strains greatly improved the growth and proline contents in *Glycine max* under salt stress. Similarly, inoculation of ABA-producing endophytic bacteria significantly enhanced salinity stress tolerance in *O. sativa* by modulating endogenous hormone and upregulating essential amino acids (e.g., glutamic acid, aspartic acid, phenylalanine, proline, and cysteine; Shahzad et al., 2017). Sadeghi et al. (2012) observed that inoculation of IAA and siderophore producing *Streptomyces* isolate significantly enhanced the growth and development of *T. aestivum*, as well as the concentration of N, P, Fe, and Mn in plant shoots in normal and saline soil. The findings suggest that *Streptomyces* isolate can be used as biofertilizers in saline soils. Recently, Khan et al. (2017) reported that the application of plant growth-promoting endophytic bacteria (PGPE) and jasmonic acid enhanced the growth of *S. pimpinellifolium* when exposed to salinity stress. The findings suggest that the salinity tolerance ability of PGPE could be attributed to the existence of glutathione-related genes in their genome.

#### Role of ACC Deaminase Producing PGPM in Abiotic Stress Tolerance

Many aspects of plant growth are regulated by ethylene concentrations and ethylene biosynthesis is tightly controlled by transcriptional and post-transcriptional factors, which are mediated by biotic and abiotic stresses (e.g., drought and salinity; Hardoim et al., 2008). The phytohormone ethylene regulates plant homeostasis in response to stress conditions, leading to reduced plant growth and development. ACC deaminase producing bacteria can modulate plant ethylene levels through cleaving ACC into *α*-ketobutyrate and ammonia, therefore, ameliorating stress and promoting plant growth under adverse conditions (Glick et al., 2007). ACC deaminase producing *Achromobacter piechaudii* ARV8 conferred IST against salt in *S. lycopersicum* (Mayak et al., 2004). Under drought conditions, ACC deaminase producing PGPB *A. piechaudii* ARV8 greatly enhanced the seedling fresh and dry weight of *L. esculentum* and *C. annuum* while reduced the production of ethylene (Mayak et al., 2004). It was found that only wild type ACC deaminase-containing PGPB (not mutant PGPB that lack ACC deaminase) could protect plants from ethylene-induced growth inhibition, regardless of whether those bacteria are rhizobacteria or endophytes in nature (Forni et al., 2017).

#### Microbial Exopolysaccharide Production

The dynamic and complex interactions between microbes, plant roots, and soils in the rhizosphere can alter the soil physicochemical and structural properties (Ma et al., 2016). The extracellular polysaccharides produced by soil microorganisms can bind soil particles together to form micro‐ and/or macroaggregates, where plant roots, bacteria, fungal hyphae fit in the pores between microaggregates and are involved in macroaggregate stabilization (Naseem et al., 2018). The EPS producing bacteria were found to enhance the plant tolerance to drought and salinity stresses due to their ability to optimize soil structure (Sandhya et al., 2009; Naseem et al., 2018). Recently, Khan and Bano (2019) demonstrated that EPS produced by PGPB consortia positively affected drought tolerance and plant growth through the improvement of soil moisture contents. In the rhizosphere soil, bacterial EPS form a rhizosheath around the roots and hence protect plant host from desiccation for a longer period. Under salinity stress, EPS may bind to cations (e.g., Na^+^), making it unavailable to plants for their uptake. The co-inoculation of *Rhizobium* and *Pseudomonas* resulted in increased proline production along with decreased electrolyte leakage, maintenance of leaf relative water content, and selective uptake of K^+^, therefore, eventually improving salt tolerance in *Z. mays* (Bano and Fatima, 2009).

#### Alteration of the Antioxidant Defense System

As mentioned above, abiotic stress such as high salinity or drought induces overproduction of ROS, leading to altered cellular redox homeostasis. The elevated ROS level causes inactivation of membrane-bound proteins, diminished membrane fluidity, DNA damage, inhibition of protein synthesis, and enzymatic activities. There is substantial evidence indicating that PGPM inoculated plants can survive under abiotic stress-induced oxidative stress by manipulation of antioxidant enzymes (Kim et al., 2014). For instance, Pedranzani et al. (2016) found that the application of AMF *Rhizophagus irregularis* improved the physiological performance of *Digitaria eriantha* under drought, salinity, and cold stresses through the upregulation of antioxidant enzyme activity (e.g., CAT and APX) and jasmonate synthesis. Recently, Ma et al. (2017) reported that the application of plant growth-promoting endophytic bacterium ASS1 stimulated the activity of CAT and SOD under various stress conditions (e.g., drought, multi-metals, and drought + multi-metals). Although PGPM inoculated plants have been proved to mitigate the oxidative damage, the underlying mechanisms behind alterations in antioxidant enzyme activities that caused by PGPM are scarcely known. Many factors, such as host plant species, PGPM type, and strain, as well as type, degree, and duration of abiotic stress can be responsible for such alterations in enzymatic levels.

#### Microbial Osmolytes Production

Plant growth-promoting microorganisms can produce compatible osmolytes in response to drought or salinity stress, which act synergistically with osmolytes (e.g., proline, trehalose, and polyamines) secreted from plants and, thus, promote plant growth and development (Paul et al., 2008). The capacity to accumulate proline under stress conditions has been greatly correlated with stress tolerance in plants (Claussen, 2005). Under drought and salinity stresses, proline shows great potential to adjust cytosolic acidity and diminish lipid peroxidation by directly scavenging ROS and stabilizing proteins and membranes (Gill and Tuteja, 2010). There have been numerous reports implicating that plants inoculated with PGPM manifest increased proline content, which helps plants to cope with drought and salinity stress (Bharti et al., 2014; (Shintu and Jayaram, 2015). However, it is still not clear whether it is absorbed from rhizosphere soils or due to the upregulation of the proline biosynthesis pathway. On the contrary, several studies observed that the inoculation of PGPB decreased the proline content in plants exposed to drought and salinity stresses (Ma et al., 2017, 2019a; Singh and Jha, 2017). This is because PGPM may counteract the adverse effects of drought and salinity by inducing the regulation of osmotic balance and maintaining the bioenergetics of plant cells.

Moreover, as a highly sable glucoside, trehalose plays an important role in diminishing the damage to plant cells from drought and salinity. Rodriguez Salazar et al. (2009) observed that inoculation of *Z. mays* with *Azospirillum brasilense* overexpressing trehalose biosynthetic genes increased trehalose accumulation, consequently conferring drought tolerance or osmotolerance. This may be attributed to the ability of trehalose to stabilize membranes and proteins. Recently, a higher accumulation of trehalose was found in mycorrhizal plants under salt stress compared to non-mycorrhizal plants. This can be due to AMF-stimulated enhanced activities of enzymes responsible for the biosynthesis of trehalose (e.g., trehalose-6-phosphate synthase and trehalose-6-phosphate phosphatase) and lower activity of trehalose degrading enzyme (e.g., trehalase; Garg and Pandey, 2016).

Polyamines are biogenic amines having aliphatic nitrogen structure that exist in almost all organisms and are widely implicated in diverse plant growth and development processes, such as cell division and differentiation, root elongation, floral development, fruit maturation, senescence, programmed cell death, and DNA replication, transcription and translation (Alcazar et al., 2011). Cassan et al. (2009) found that inoculation of cadaverine (polyamine)-producing *A. brasilense* Az39 significantly promoted the root growth of *Oryza* seedlings under osmotic stress (Cassan et al., 2009). Recently, a pot experiment was conducted by Zhang et al. (2019b) to evaluate the role of AMF in root polyamine homeostasis, activities, and gene expression of polyamine-related synthesizing and degrading enzymes in *Poncirus trifoliata* under drought stress. The results show that mycorrhizal application induced higher putrescine and cadaverine with higher activity of polyamine catabolic enzymes and putrescine synthases under drought stress, demonstrating that mycorrhizas can improve plant drought tolerance through modulation of polyamine metabolism.

## Conclusion and Future Perspectives

Considering the global climate change scenario, the main obstacle to global food security is sustained loss of crops due to abiotic stresses (particularly drought and salinity). In the past decades, great progress has been made in understanding how abiotic stresses affect plant growth and yield, and how the plant respond/adapt to these stresses. The duration and severity of drought or salinity exposed have undoubtedly pivotal roles in examining how plants respond to these stresses as elucidated in **Figure 1**.

In terms of drought, stomata close progressively along with a parallel reduction in water-use efficiency and net photosynthetic activity. Apart from other parameters, the alterations in photosynthetic pigments are closely correlated with drought tolerance. Self-protective responses to the stress at the leaf level must then be triggered quickly to protect the photosynthetic machinery from being irreversibly damaged. Scavenging of ROS by enzymatic and non-enzymatic systems, cell membrane stability, expression of stress-responsive genes, and proteins are essential mechanisms of drought tolerance. Moreover, metabolite adjustments strongly contribute to drought adaptation, particularly polyphenols, lipophilic compounds, and some TCA cycle metabolites are involved in defense and protection, while other compounds, such as carbohydrates, amino acids, and polyols, contribute to osmoregulation.

Plant response to salinity follows a biphasic model, wherein an early phase shows a similarity with drought (osmotic stress) and in the long-term induce ion toxicity. In the first phase, growth falls significantly, and stomata closure occurs in response to water potential decline. In the second phase, ions accumulations, particularly Na^+^, affects photosynthetic components such as enzymes and pigments, and increase oxidative stress. ROS play a dual role in salt stress response, functioning as toxic by-products of stress, as well as a signal molecule activating several pathways leading to protective responses that are also common to drought stress.

Plant-associated microorganisms can be considered as major components of the ecosystems and play an essential role in improving plant adaptation/evolution to climatic stresses (such as drought and salinity). In this regard, microbes can rescue plants from the negative consequences of drought and salinity through various mechanisms, such as solubilization of nutrients (N, P, K, and Fe), IST, and production of phytohormones (IAA, cytokinin, ABA, and GA), EPS, and ACC deaminase. Given the fundamental understanding of these mechanisms involved in plant-PGPM interactions, it is expected that the practical use of PGPM in agricultural fields will grow dramatically to improve plant survival to environmental changes. Nevertheless, it is not clear whether the mechanisms involved in PGPM induced amelioration of drought stress are different from those of salinity stress. Further research is required to provide evidence of underlying similarities and differences in microbe induced drought and salinity tolerance, basing on innovations in mirroring microbial interactions found in nature.

## Author Contributions

YM developed the ideas and wrote and revised the manuscript. MD wrote and revised the manuscript. HF was the project sponsor, and revised the manuscript. All authors contributed to the article and approved the submitted version.

### Conflict of Interest

The authors declare that the research was conducted in the absence of any commercial or financial relationships that could be construed as a potential conflict of interest.
